# A Mathematical Model of the Olfactory Bulb for the Selective Adaptation Mechanism in the Rodent Olfactory System

**DOI:** 10.1371/journal.pone.0165230

**Published:** 2016-12-19

**Authors:** Zu Soh, Shinya Nishikawa, Yuichi Kurita, Noboru Takiguchi, Toshio Tsuji

**Affiliations:** 1 Department of System Cybernetics, Institute of Engineering, Hiroshima University, Higashi-Hiroshima, Japan; 2 Department of System Cybernetics, Graduate School of Engineering, Hiroshima University, Higashi-Hiroshima, Japan; 3 Division of Material Sciences, Graduate School of Natural Science and Technology, Kanazawa University, Kanazawa, Japan; Monell Chemical Senses Center, UNITED STATES

## Abstract

To predict the odor quality of an odorant mixture, the interaction between odorants must be taken into account. Previously, an experiment in which mice discriminated between odorant mixtures identified a selective adaptation mechanism in the olfactory system. This paper proposes an olfactory model for odorant mixtures that can account for selective adaptation in terms of neural activity. The proposed model uses the spatial activity pattern of the mitral layer obtained from model simulations to predict the perceptual similarity between odors. Measured glomerular activity patterns are used as input to the model. The neural interaction between mitral cells and granular cells is then simulated, and a dissimilarity index between odors is defined using the activity patterns of the mitral layer. An odor set composed of three odorants is used to test the ability of the model. Simulations are performed based on the odor discrimination experiment on mice. As a result, we observe that part of the neural activity in the glomerular layer is enhanced in the mitral layer, whereas another part is suppressed. We find that the dissimilarity index strongly correlates with the odor discrimination rate of mice: *r* = 0.88 (*p* = 0.019). We conclude that our model has the ability to predict the perceptual similarity of odorant mixtures. In addition, the model also accounts for selective adaptation via the odor discrimination rate, and the enhancement and inhibition in the mitral layer may be related to this selective adaptation.

## Introduction

Predicting the quality of an odor composed of multiple odorant components is a challenging problem. Even if the smell of each odorant component was known, the resultant quality of an odorant mixture may differ from the linear addition of the respective odorant qualities [1].

An interesting approach to predicting odorant quality is that proposed by Haddad *et al*., in which an odorant quality space was derived from about 1400 kinds of odorant descriptor using principal analysis [2]. Their group also applied the developed method to predict the pleasantness of an odorant [3]. Our group proposed a model that can predict glomerular activity patterns from the chemical structure of an odorant [4], and found that the similarity indices between the activity patterns of rats correlates with the perceptual similarity of humans [5]. Hence, the prediction of the quality of a single odorant is gradually becoming a reality, although more accurate prediction is desirable. Recently, odor quality predictions for odorant mixtures have been studied. Snitz *et al*. used odorant descriptors to form a chemical space and proposed a distance measure between the odorant mixtures that enables the perceptual similarity to be predicted [6]. Haddad *et al*. proposed a method that can convert artificial sensor responses into the pleasantness of both odorants and odorant mixtures [7].

In addition to the techniques described above, this paper focuses on the brain expression of odors evoked on the olfactory bulb, including the odor maps on the glomerular layer [8–13], to predict the perceptual similarity of odorant mixtures. Previous studies [14, 15] have revealed the close relationship between neural activity and odor quality, and neural interaction in the olfactory system can change the characteristics of odor perception. For example, Youngentob *et al*. reported that the glomerular activity patterns can predict the perceptual similarity of odorants [14], and the same holds for odorant mixtures according to Grossman *et al*. [15]. In addition, through odor discrimination experiments on mice, we have found that selective adaptation contributes significantly to perceptual similarity [16]. That is, after associating the reward with a mixture of three odorants, the mice adapted to certain components in the mixture, leading to trouble distinguishing between the full mixture and certain subsets of these odorants. The same phenomenon was observed in an experiment on humans, who used selective adaptation to improve the reliability of identification of odor components in a mixture. This is because humans cannot identify the respective odor characteristics of more than three odorant components [17]. This selective adaptation function may be consistent with measurement results given by Giraudet *et al*. [18], which suggest that, in most cases, the response of the mitral cells to a binary mixture is dominated by one of the components.

Although selective adaptation in the olfactory system has been observed, its application to the prediction of perceptual similarity has not been studied. This paper thus proposes a mathematical model based on previous olfactory models [19, 20] that attempted to simulate the selective adaptation toward odorant components. We use the proposed model to generate neural activity patterns in the olfactory bulb, including the glomeruli, mitral cells, and granular cells, and compare the activity patterns of mitral cells evoked by different odorant mixtures. The similarity between odorant mixtures is identified by examining the correlation between activity patterns, and the results are compared to the perceptual similarity sensed by mice. The results show that the proposed model is capable of predicting the discrimination rate of mice with a strong correlation (*r* > 0.8, *q* < 0.05). This suggests that the model is capable of predicting perceptual similarity considering the selective adaptation mechanism.

The remainder of this paper is organized as follows. Section 2 explains the structure of the proposed model and our parameter adjustment algorithm, as well as the simulation procedure. Section 3 presents the simulation results and related discussion, and Section 4 states our conclusions. An odor discrimination experiment conducted on mice is described in S1 Appendix. S1 Appendix also lists and explains the symbols used in the model.

## Materials and Methods

### Olfactory model

**Fig 1:**
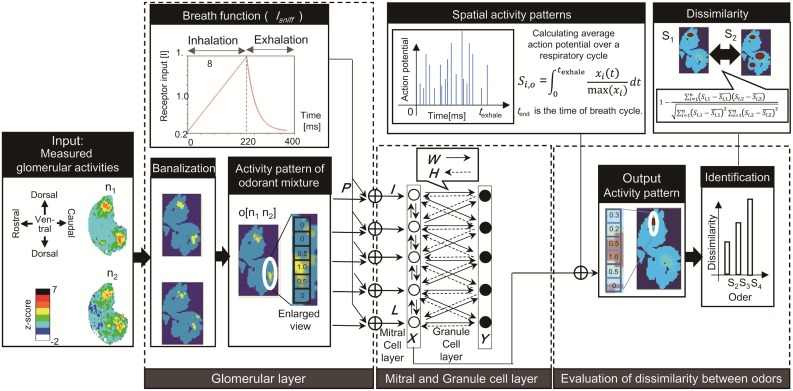
Schematic diagram of the proposed olfactory model. The model consists of a glomerular layer, mitral and granular layer, and a dissimilarity evaluation module. The model takes the glomerular activity patterns of odorants composing an odor as input, and considers respiration cycles to simulate the glomerular response to odorant mixture. The neural activity in mitral and granular cells is simulated based on the models proposed in a previous study [20, 21]. The dissimilarity evaluation module defines a dissimilarity index *E* and compares the activity patterns evoked in the mitral layer by different input odorant mixtures.

The proposed model is composed of a glomerular layer and a mitral cell/granular cell layer, as shown in Fig 1. The model takes measured glomerular activity patterns as input, and outputs a dissimilarity index between odors. The parameters and symbols used in the model are summarized in Tables A-E in S1 Appendix.

#### Glomerular Layer

**Fig 2:**
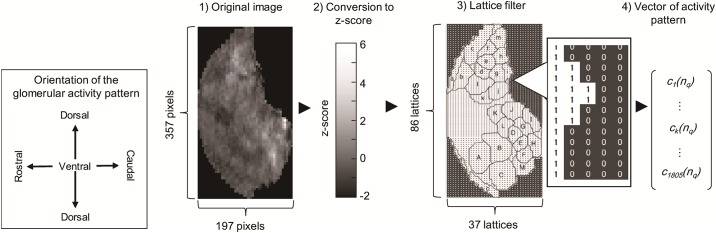
Method of generating the model input from measured glomerular activity patterns provided by a web database (http://gara.bio.uci.edu/) (adapted from [5]). The original image of the glomerular activity pattern (left) is composed of 357 × 197 pixels, and the grayscale of each pixel corresponds to the activity strength. The original image is divided into 1805 lattices, approximately equal to the actual number of glomeruli distributed on the olfactory bulb. The average activity strength is calculated for each pixel and converted into a vector representing an activity pattern.

The glomerular layer, which is composed of 1805 glomerular units, takes each measured glomerular activity pattern of the odorant components as input. The number of glomerular units is consistent with the actual number of glomeruli distributed on the olfactory bulbs of mice [21]. It then generates the glomerular activity evoked by an odorant mixture taking the respiratory cycle into account. The input of the *i*-th glomerular unit *c*_*i*_(*n*_*q*_) generated by odorant *n*_*p*_ is determined by the following procedure (*cf*. Fig 2), where *q* is the index of the odorant component.

1. Obtain a whole olfactory bulb surface image of the glomerular activity pattern corresponding to odorant *n*_*q*_ from a web database (http://gara.bio.uci.edu/).
2. Based on the procedure used in our previous study [5], the image file of the activity pattern is divided into 1805 lattices corresponding to the glomerular units. First, each pixel of the activity pattern image is converted into a z-score corresponding to its activation level based on the color bar given by the database. A lattice filter is then adapted to divide the activity pattern into 1805 lattices.
3. Calculate the average z-score of each lattice and determine the input *c*_*i*_(*n*_*q*_).

The above procedure provides the input vector $C\left(n_{q}\right)=[c_{1}\left(n_{q}\right),\ldots \;,c_{k}\left(n_{q}\right),\ldots \;,c_{1805}\left(n_{q}\right)]\in {\mathbb{R}}^{1805\times 1}$ for odorant component *n*_*q*_.

The output *I*_siniff,i_(*t*) of the *i*-th glomerular unit at time *t* is given by the following equations, which are based on a respiratory function that originated from measurement data of a rabbit and was employed in the olfactory model proposed by Li *et al*. [19] (*cf*. Fig 1: Breath Function):

$$I_{\text{sniff},i}\left(t\right)=\{\begin{array}{ll} P_{i}\left(o_{\gamma }\right)\left(t-t_{\text{inhale}}\right)+I_{\text{sniff},i}\left(t_{\text{inhale}}\right), & \text{if}\;0≦t≦t_{\text{inhale}},\\ I_{\text{sniff},i}\left(t_{\text{exhale}}\right)\text{exp}\left(\frac{-\left(t-t_{\text{exhale}}\right)}{\tau_{\text{exhale}}}\right), & \text{if}\;t_{\text{inhale}}<t≦t_{\text{exhale}}, \end{array}$$ (1)

where *t*_inhale_ = 220 [ms], *t*_exhale_ = 400 [ms], *τ*_exhale_ = 33 [ms] are the inhalation duration, exhalation duration, and time constant of exhalation, respectively [19]. In addition, $P=\left[P_{1}\left(o_{\gamma }\right),\ldots \;,P_{k}\left(o_{\gamma }\right),\ldots \;,P_{1805}\left(o_{\gamma }\right)\right]\in {\mathbb{R}}^{1805\times 1}$ denotes the maximum output of a glomerular unit to odor *o*_*γ*_, and is calculated by Eq (2). This equation was derived under the assumption that a glomerular activity pattern of an odorant mixture is the linear combination of its odorant components [10]. Note that this is a simplified formulation, because Grossman *et al*. [15] reported that a simple linear addition is not always applicable.

$$P_{i}\left(o_{\gamma }\right)=\frac{1}{k}\sum \begin{array}{l} k\\ q=1 \end{array}f\left(c_{i}\left(n_{\text{q}}\right)\right),\;f\left(c_{i}\left(n_{q}\right)\right)=\{\begin{array}{ll} 1, & \text{if}\;\;\;c_{i}\left(n_{p}\right)≧\theta \\ 0, & \text{if}\;\;\;c_{i}\left(n_{p}\right)<\;\theta \end{array},$$ (2)

where *k* is the number of odorant components in an input odor, *n*_*q*_ is the odorant component in odorant mixture *o*_*γ*_, and *θ* is a threshold variable. The threshold function *f*(·) was applied to enhance the contrast, because the 2-deoxyglucose (2-DG) method can blur the spatial response. The output *P*_*i*_(*o*_*γ*_) of the *i*-th glomerular unit is sent to the mitral unit.

#### Mitral and Granular layer

The mitral and granular layer was derived from the Li–Hopfield model [19] and the Erdi model [20]. These models, which can simulate the neural dynamics caused by the interaction between mitral and granular cells, can be written as follows:

$$I_{i}=I_{\text{sniff},\text{i}}+I_{\text{background}}$$ (3)

$$\left\{ \begin{array}{l} \dot{X}=-HG_{Y}-\frac{1}{\tau_{x}}X+c\sum \begin{array}{l} N\\ j=1 \end{array}LG_{X}+I\\ \dot{Y}=WG_{X}-\frac{1}{\tau_{y}}Y+I_{c} \end{array}\right.$$ (4)

Where $X=[x_{1},\ldots \;,x_{k},\ldots \;,x_{1805}]\in {\mathbb{R}}^{1805\times 1}$ and $Y=[y_{1},\ldots \;,y_{k},\ldots \;,y_{1805}]\in {\mathbb{R}}^{1805\times 1}$ are the internal states of the mitral and granular cells, respectively, $I=\left[I_{1},\ldots ,I_{k},\ldots ,\;I_{1805}\right]\in {\mathbb{R}}^{1805\times 1}$ is the input from the glomeruli, *I*_background_ is the background noise, *I*_c_ is the excitatory input from the olfactory cortex, and *τ*_*x*_ = *τ*_*y*_ = 7 [ms] are time constants [20]. $H,L,G\in {\mathbb{R}}^{1805\times 1805}$ are synapse connection matrices, where ***H*** represents the connection from granular cells to mitral cells, ***L*** represents that between mitral cells, and ***G*** represents that from mitral cells to granular cells. In addition, $G_{Y}=[g_{y}\left(y_{1}\right),\ldots \;,g_{y}\left(y_{k}\right),\ldots \;,g_{y}\left(y_{1805}\right)]\in {\mathbb{R}}^{1805\times 1}$ corresponds to the membrane potentials of mitral and granular cells, respectively. Based on the Li–Hopfield model, the membrane potentials are calculated by the following equations [19]:

$$g_{x}\left(x_{i}\right)=\{\begin{array}{lll} S_{x}^{\prime }+S_{x}^{\prime }\text{tanh}\left(\frac{x_{i}-\zeta }{S_{x}^{\prime }}\right), & \text{if}\;x<\zeta , & S_{x}^{\prime }=0.14\\ S_{x}^{\prime }+S_{x}\text{tanh}\left(\frac{x_{i}-\zeta }{S_{x}}\right), & \text{if}\;x≧\zeta , & S_{x}=1.4 \end{array},$$ (5)

$$g_{y}\left(y_{i}\right)=\{\begin{array}{lll} S_{y}^{\prime }+S_{y}^{\prime }\text{tanh}\left(\frac{y_{i}-\zeta }{S_{y}^{\prime }}\right), & \text{if}\;y<\zeta , & S_{y}^{\prime }=0.29\\ S_{y}^{\prime }+S_{y}\text{tanh}\left(\frac{y_{i}-\zeta }{S_{y}}\right),\; & \text{if}\;y≧\zeta , & S_{x}=2.9 \end{array},$$ (6)

where the threshold *ζ* is set to 1.0 based on the Erdi model [20]. As several tens of mitral cells typically receive excitatory input from the same glomerulus [22], they can be considered to form a column [23]. Thus, a mitral unit represents a mitral cell column, and each mitral unit is connected to one glomerular unit. In the same manner, the granular units represent groups of granular cells. Thus, in the proposed model, the mitral units and granular units share the same spatial distribution as the glomerular units.

**Fig 3:**
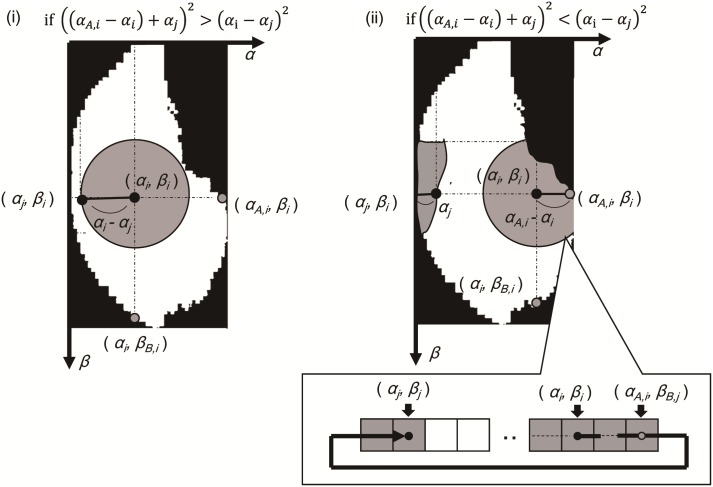
Configuration of synapse connections. The figure represents the connection range of a mitral or a granular unit. A unit at the center of the grey circle is connected to all units within a range of the circle. If the connection range exceeds the limit, it is folded back to the other end, considering the bulbous shape of the olfactory bulb, using Eqs (7)–(12).

To determine the synapse connection matrices, we consider each type of neuron to form a layer on a spherical surface in the olfactory bulb. First, a unit is placed on a two-dimensional *α-β* coordination, as shown in Fig 3. Each mitral unit is then connected to the other mitral units within a distance of *ζ*_*m*_ units based on the actual connection structure of mitral cells [18]. In the same manner, each granular unit is connected to the mitral units within a distance of *ζ*_*g*_ units. If this distance exceeds the extent of the two-dimensional surface, the connection is folded back to the opposite end, considering the spherical surface of the olfactory bulb shown on the right of Fig 3. The gray shadow in Fig 3 is an example of the connection range from the *i*-th unit located at (*α*_*i*_, *β*_*i*_). To achieve such a connection, the following equations are used to determine the synapse connection matrices:

$$d_{\alpha \left(i,j\right)}=\text{min}\left({\left(\text{min}\left(\alpha_{A,i}-\alpha_{i},\;\alpha_{i}\right)-\text{min}\left(\alpha_{A,i}-\alpha_{j},\;\alpha_{j}\right)\right)}^{2},{\left(\alpha_{i}-\alpha_{j}\right)}^{2}\right)$$ (7)

$$d_{\beta \left(i,j\right)}=\text{min}\left({\left(\text{min}\left(\beta_{B,i}-\beta_{i},\;\beta_{i}\right)+\text{min}\left(\beta_{B,i}-\beta_{j},\;\beta_{j}\right)\right)}^{2},{\left(\beta_{i}-\beta_{j}\right)}^{2}\right)$$ (8)

$$d_{\left(i,j\right)}=\sqrt{d_{\alpha \left(i,j\right)}^{2}+d_{\beta \left(i,j\right)}^{2}}$$ (9)

$$H_{0\left(i,j\right)}=\{\begin{array}{ll} \frac{R_{i,j}}{\zeta_{m}}, & \text{if}\;0<d_{\left(i,j\right)}<\zeta_{m}\\ 0, & \text{if}\;\zeta_{m}<d_{\left(i,j\right)} \end{array}$$ (10)

$$W_{0\left(i,j\right)}=\{\begin{array}{ll} \frac{R_{w,i}}{\zeta_{g}}, & \text{if}\;0<d_{\left(i,j\right)}<\zeta_{g}\\ 0, & \text{if}\;\zeta_{g}<d_{\left(i,j\right)} \end{array}$$ (11)

$$L_{0\left(i,j\right)}=\{\begin{array}{ll} R_{l,i}, & \text{if}\;0<d_{\left(i,j\right)}<\zeta_{g}\\ 0, & \text{if}\;\zeta_{g}<d_{\left(i,j\right)} \end{array}$$ (12)

where *d*_*α*(*i*,*j*)_ and *d*_*β*(*i*,*j*)_ denote the distance between two units along the *α*-axis and *β*-axis, respectively. $H_{0}=[H_{0}{}_{\left(1,1\right)},\ldots \;,H_{0}{}_{\left(j,k\right)},\ldots \;,H_{0}{}_{\left(1805,1805\right)}]\in {\mathbb{R}}^{1805\times 1805}$ is the synapse connection matrix from granular units to mitral units, $W_{0}=[W_{0}{}_{\left(1,1\right)},\ldots \;,W_{0}{}_{\left(j,k\right)},\ldots \;,W_{0}{}_{\left(1805,1805\right)}]\in {\mathbb{R}}^{1805\times 1805}$ is that from mitral units to granular units, and $L_{0}=[L_{0}{}_{\left(1,1\right)},\ldots \;,L_{0}{}_{\left(j,k\right)},\ldots \;,L_{0}{}_{\left(1805,1805\right)}]\in {\mathbb{R}}^{1805\times 1805}$ is that between mitral units. Note that, in Eqs (10) and (11), *R*_*i*,*j*_, *R*_*w*,*i*_, *R*_*l*,*i*_ are random numbers selected from the normal distribution *N*(1.0,0.05), and each connection parameter is divided by the connection range parameters *ζ*_*m*_ or *ζ*_*g*_ so that the input strength to a unit is independent of the connection range parameters.

#### Dissimilarity Evaluation Part

The mitral cells transfer neural activity to the olfactory cortex, and higher brain functions judge the odor quality based on individual experiences [23]. In the proposed model, the dissimilarity evaluation simulates this role by calculating a dissimilarity index based on the Pearson correlation between the spatial patterns of the mitral layer evoked by different odors. The action potential of each mitral unit responding to an odor *o*_γ_(*n*_*q*_) composed of odorants *n*_*q*_ is averaged over the respiration duration (*t*_exhale_ = 400 [ms]) using the following equation to give the spatial activity pattern of the mitral layer $S_{o_{\gamma }}=\left[S_{1,o_{\gamma }},\ldots \;,S_{k,o_{\gamma }},\ldots \;,S_{1805,o_{\gamma }}\right]\in {\mathbb{R}}^{1805\times 1}$.

$$S_{i,o_{\gamma }}=\int \begin{array}{l} t_{\text{exhale}}\\ 0 \end{array}\frac{x_{i}\left(t\right)}{\text{max}\left(x_{i}\right)}dt$$ (13)

The dissimilarity index between arbitrary odors *o*_1_ and *o*_2_ is then calculated by the following equation, which reflects the ease of discrimination, that is, the discrimination rate obtained from the mice experiment.

$$E=1-\frac{\sum_{i=1}^{n}\left(S_{i,1}-\bar{S_{i,1}}\right)\left(S_{i,2}-\bar{S_{i,2}}\right)}{\sqrt{\sum_{i=1}^{n}{\left(S_{i,1}-\bar{S_{i,1}}\right)}^{2}}\sqrt{\sum_{i=1}^{n}{\left(S_{i,2}-\bar{S_{i,2}}\right)}^{2}}}$$ (14)

Therefore, the dissimilarity index *E* can be compared to the observed discrimination rate to test whether the model can predict the perceptual similarity exhibited by mice, from which the selective adaptation mechanism in the olfactory system was identified.

### Simulation procedure

In our simulations, the synapse connection matrix ***H*** was adjusted using a Hebbian learning rule [20, 24] based on the experimental procedure applied to mice [16], and then the dissimilarity index obtained from the model was compared to the experimentally observed discrimination rate. We consider the proposed model to correctly account for selective adaptation if it can predict the perceptual similarity of mice. This section describes the simulation procedure, including a parameter adjustment and comparison method between the simulations and experiments.

#### Configurations

The majority of the parameters included in the proposed model were determined based on the Li–Hopfield model [19] and the Erdi model [20], as shown in Tables C and D in S1 Appendix. The newly introduced parameters in the proposed model are the threshold *θ* that determines the activity pattern of the glomerular layer, and *ζ*_*m*_, *ζ*_*g*_ that determine the synapse connection range of mitral units and granular units, respectively. The threshold parameter was set to *θ* = 0.60 to enhance the strongly activated part corresponding to a z-score greater than 2 measured by the 2-DG method. The synapse connection range parameters were set to *ζ*_*m*_ = 4 and *ζ*_*g*_ = 15 under the assumption that granular cells have a wider influential range than the mitral cells [25]. In addition, each component of the synapse connection matrices ***H***, ***L***, ***W*** was determined by Eqs (10)–(12), and 20 sets of initial connection matrices were generated to perform the following simulations. The random numbers *R*_*i*,*j*_, *R*_*w*,*i*_, *R*_*l*,*i*_ used to generate different connection matrices allow us to test the robustness of prediction ability against different configurations of initial values.

#### Prediction of perceptual similarity

STEP 1: Simulation of conditioning training
  Based on the mice experiment [16], the rewarded odor ([IA, EB, Ci], *cf*. Table F in S1 Appendix) was input to the proposed model. Assuming that selective adaptation is caused by the interaction between mitral cells and granular cells, the proposed model used the following Hebbian rule [20, 24] to learn the synapse connection matrix ***H***:
  $${\dot{H}}_{ij}\left(t\right)=-\eta_{1}H_{ij}^{2}+\eta_{2}g_{x}\left(x_{i}\left(t\right)\right)g_{y}\left(y_{j}\left(t\right)\right)$$ (15)
  where the learning rates were set to *η*_*1*_ = 10^−5^ and *η*_*2*_ = 10^−3^ [19], and the adjustment was terminated when the connection parameters converged to constant values. Here, the activity pattern of the mitral layer when rewarded odor [IA, EB, Ci] was input after parameter learning is denoted as $S_{1}=[S_{1,1},\ldots \;,S_{k,1},\ldots \;,S_{1805,1}]\in {\mathbb{R}}^{1805\times 1}$.
STEP 2: Odor discrimination simulation
  Based on the mice experiments, five discrimination target odors (except the rewarded odor [IA, EB, Ci], *cf*. Table F in S1 Appendix) were input to the model with adjusted synapse connection matrix ***H***, and each activity pattern obtained was represented by $S_{2}=\left[S_{1,2},\ldots \;,S_{k,2},\ldots \;,S_{1805,2}\right]\in {\mathbb{R}}^{1805\times 1}$. Activity patterns ***S***_***1***_ evoked by the rewarded odor and ***S***_***2***_ evoked by the discrimination target were substituted into Eq (14), and the dissimilarity index *E* was calculated. As the dissimilarity index corresponds to the discrimination rate obtained from the experiments on mice, we computed the Pearson correlation between the dissimilarity indices and the discrimination rate.

#### Parameter analysis

To test the dependency of the prediction accuracies on the connection structure in the mitral and granular layers, various connection ranges *ζ*_*m*_ and *ζ*_*g*_ were iteratively searched. Based on a previous study [25, 26], we assumed that granular cells have a wider range of influence than the mitral cells, and searched for parameters in the range 3 ≦ *ζ*_*m*_ ≦ 7, 13 ≦ *ζ*_*g*_ ≦ 17. We then determined the combination of *ζ*_*m*_ and *ζ*_*g*_ that yielded the highest correlation between the dissimilarity index *E* and discrimination rate of rats. We also investigated the changes in the activity patterns of the mitral layer and synapse connection parameters in ***H*** along with the Hebbian learning.

## Results and Discussion

### Results

**Fig 4:**
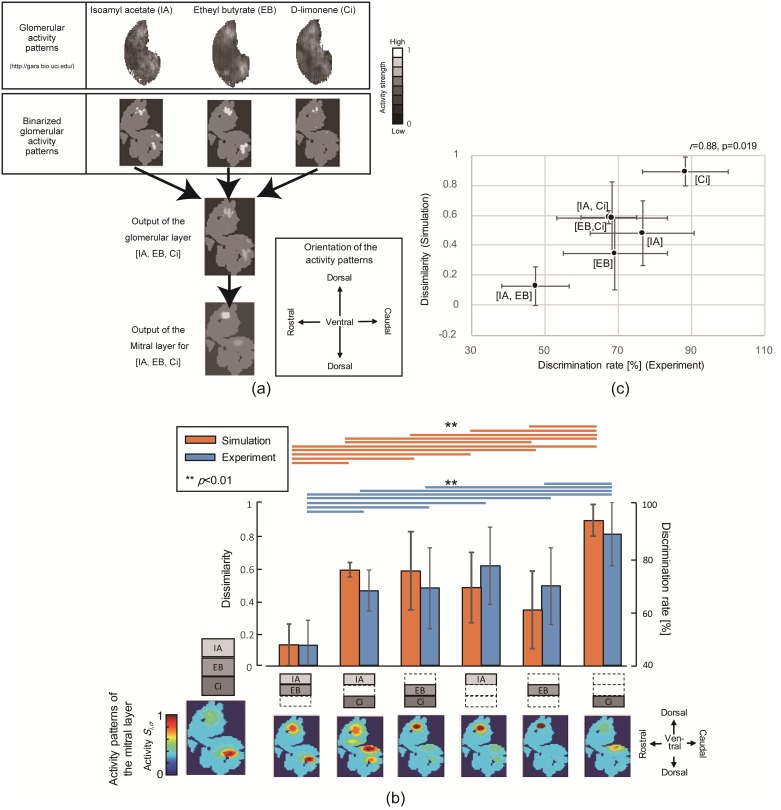
Prediction of perceptual similarity for odors composed of IA, EB, and Ci. (a) Output from the glomerular layer for odorant input [IA, Ci, EB]. The figure shows the steps taken to generate output for the glomerular layer. The activity strength is represented in grayscale, where whiter pixels correspond to higher activity. The uppermost row shows the glomerular activity pattern for the odorant composing odor [IA, EB, Ci] obtained from Johnson *et al*. [27–31]. The middle row shows the binarized activity patterns given by adapting Eq (2) after dividing the pixels into 1805 lattices and generalizing the activity strength into the range [0, 1]. The third row shows the output from the glomerular layer, and the bottom row shows that from the mitral layer generated by the procedure described in Section 2. (b) Comparison between discrimination rates of mice obtained from experiments and dissimilarity obtained from simulations (*ζ*_*m*_ = 4, *ζ*_*g*_ = 15). The figure compares the dissimilarity index *E* obtained from the simulation and discrimination rate for each odor. The orange bars denote simulation results and the blue bars represent the experimental results. The error bars added to the experimental results address the standard deviation in 10 mice, and the error bars added to the simulation results correspond to the standard deviation of 20 sets of synapse connection parameters. Orange and blue lines above the bars denote a significant difference of *p*<0.01 between odor pairs. Orange lines represent multiple comparison results from the simulation, and blue lines represent that of experiments on mice. (c) Scatter plot between discrimination rates of mice obtained from experiments and dissimilarity index E obtained from simulations. The figure shows a scatter plot between the dissimilarity index and discrimination rate. The error bars correspond to those in (b).

Fig 4 shows the simulation results for an odor set composed of odorants Isoamyl acetate (IA), Etyhl butyrate (EB), and Citral (Ci). Fig 4(a) shows the transformation from glomerular activity patterns of IA, EB, and Ci into the input of the glomerular layer based on the procedure described in Section 2. Fig 4(b) compares the dissimilarity index *E* obtained from the simulation along with the discrimination rate of mice. Fig 4(c) shows a scatter plot of the dissimilarity index *E* and discrimination rate. Fig 4(b) and 4(c) confirm a strong correlation (*r* = 0.88, *p* = 0.019) between the discrimination rate of mice and the dissimilarity index *E*.

To evaluate the prediction ability of perceptual similarity, multiple testing was performed using Bonferroni’s method on the discrimination rate of mice between odor pairs. The results are shown in Fig 4(b), where blue lines above pairs of bars denote a significant difference (*p*<0.01) between the corresponding odor pairs, and in the second column of Table 1, where double asterisks represent a significant difference. Multiple testing was also adopted for the dissimilarity index *E* between odor pairs. The results are shown in Fig 4(b) and the third column of Table 1. These results show that the dissimilarity index *E* of [IA, EB] is significantly lower (*p*<0.01: green background in Table 1) than that for the other five odors ([IA Ci], [EB Ci], [IA], [EB], [Ci]), the dissimilarity index of [Ci] is significantly higher (*p*<0.01: yellow background in Table 1) than that of the other odors ([IA EB], [IA Ci], [EB Ci], [IA], [EB]), and there is little significant difference among the dissimilarity indices of the four odors [IA, Ci], [EB, Ci], [IA], and [EB] (white background in Table 1). This result is generally consistent with the discrimination rates of mice, as shown in the second column of Table 1.

**Table 1:** Difference between discrimination rates (Experiment) of odor pairs and that between dissimilarity index (Simulation).

Odor pair	Experiment	Simulation
[IA, EB]-[IA, Ci]	**	**
[IA, EB]-[EB, Ci]	**	**
[IA, EB]-[IA]	**	**
[IA, EB]-[EB]	**	**
[IA, EB]-[Ci]	**	**
[IA, Ci]-[EB, Ci]	-	-
[IA, Ci]-[IA]	-	-
[IA, Ci]-[EB]	-	**
[EB, Ci]-[IA]	-	-
[EB, Ci]-[EB]	-	-
[IA]-[EB]	-	-
[Ci]-[IA]	-	**
[Ci]-[EB]	**	**
[Ci]- [IA, Ci]	**	**
[Ci]- [EB, Ci]	**	**

**: *p*<0.01

**Fig 5:**
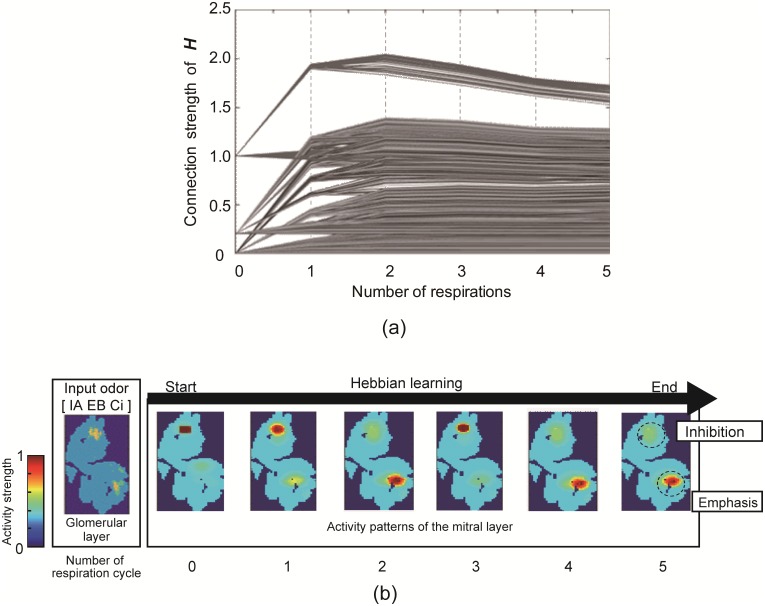
Results of Hebbian learning. (a) Convergence of the synapse connection strength of ***H***. (b) Changes of activity patterns in mitral layer. The deep red parts are the most activated and blue parts are least activated.

In the above simulations, the Hebbian learning rule [20, 24] was used to learn the synapse connection matrix ***H***. Fig 5(a) shows the parameters in ***H*** with respect to time, and Fig 5(b) shows the activity pattern of the mitral layer with respect to the respiratory cycle. Fig 5(a) confirms the convergence of the synapse connection parameters within five respiratory cycles. Fig 5(b) demonstrates that part of the input activity pattern has been enhanced, while the remainder is inhibited as the Hebbian learning evolves.

**Fig 6:**
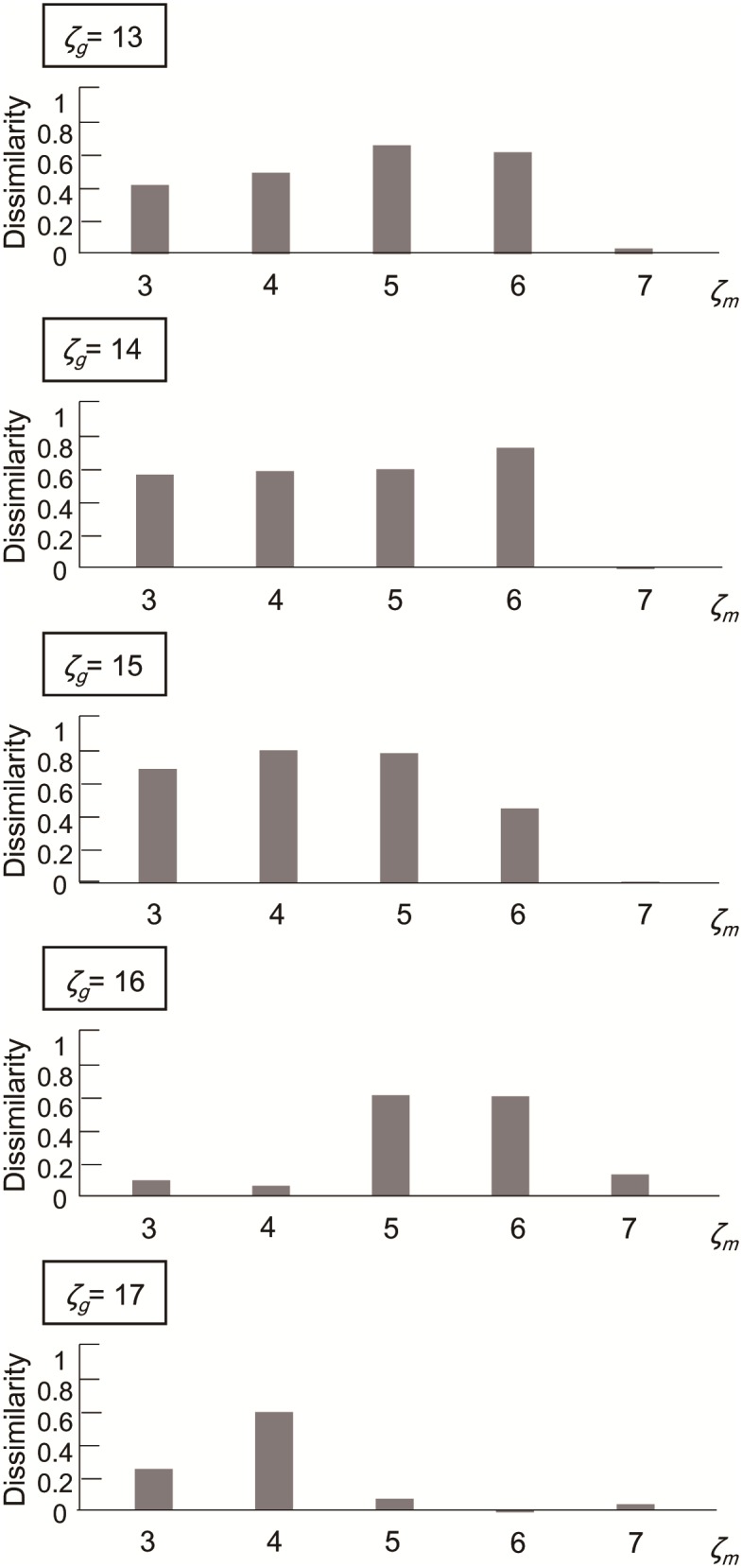
Parameter set *ζ*_*m*_, *ζ*_*g*_ and prediction accuracy. Pearson correlations between dissimilarity index *E* and discrimination rate of the mice obtained from experiments are plotted with different *ζ*_*g*_ and *ζ*_*m*_.

The parameters *ζ*_*m*_ and *ζ*_*g*_, determine the number of neighbor units connected to each mitral and granular unit, and correspond to the axonal length extended from the mitral cells and granular cells in the actual olfactory bulb, respectively. To test the dependency on the connection structure, the parameters *ζ*_*m*_ and *ζ*_*g*_, were iteratively varied over the range 3–7 and 13–17, respectively (giving a total of 25 parameter combinations). The resultant correlations between the dissimilarity index *E* and discrimination rate of mice [16] are shown in Fig 6, from which the best parameter combination was determined to be *ζ*_*m*_ = 4 and *ζ*_*g*_ = 15.

### Discussion

Fig 4(a) shows that the output of the glomerular layer for the odor [IA, EB, Ci] is a linear addition of the activity patterns of each odorant component. This representation is consistent with the measurement data provided by Belluscio and Katz [11]. The output of the mitral layer suppressed part of the activity in the glomerular layer, which may correspond to selective adaptation. Indeed, Fig 4(c) confirms that there is a strong correlation between the dissimilarity index *E* and the discrimination rate of mice (*r* = 0.88, *p* = 0.019). The mice find it difficult to distinguish between the full mixture odor [IA, EB, Ci] and the subset mixture odor [IA, EB]. These results can be interpreted as the mice learning to selectively adapt and attend to certain glomeruli, which happen to be very similar for the full mixture as well as the subsets that mice have difficulty identifying.

Fig 4 also show that accurate perceptual similarity of odors [IA, Ci], [EB, Ci], [IA Ci], [IA], and [EB] to odor [IA EB Ci] are difficult to predict, and the correlation largely depends on [IA, EB] and [Ci]. However, Table 1 demonstrates that the model can predict three levels of odor similarity: odor [IA, EB] is significantly similar to [IA, EB, Ci], odor [Ci] is significantly dissimilar to [IA, EB, Ci], and odors [IA, Ci], [EB, Ci], [IA], and [EB] are moderately similar to [IA, EB, Ci]. In this regard, the results of multiple testing presented in Table 1 indicate the robustness of the prediction ability of the model against random initial values of connection matrices ***H***, ***W***, and ***L***. The proposed model can therefore be considered to have the ability to predict the perceptual similarity between odors. In other words, as selective adaptation was observed in the odor discrimination rate of mice and the model can predict this discrimination rate, we can conclude that the model is sufficiently accurate to account for selective adaptation.

Fig 5(a) shows that the synapse connection parameters converged after Hebbian learning, and the learning step can be terminated after five respiratory cycles. Fig 5(b) shows that part of the activity pattern input from the glomerular layer to the mitral layer was enhanced, whereas the other part was suppressed. This simulation result may correspond to the measurement data reported by Giraudet *et al*. [19], which indicated that one of the binary mixture components generally dominates the activity pattern on the mitral layer. Therefore, the measurement data of Giraudet *et al*. [19] may be a neural representation of selective adaptation in the olfactory system. Further investigation into selective adaptation should therefore measure the activity of mitral cells. In addition, as the selective adaptation emerged from Hebbian learning, our approach could be used to account for the difference in perceptual characteristics caused by odor experiences.

From the results of the parameter search described in Fig 6, the connection range parameters of mitral cells and granular cells were determined to be *ζ*_*m*_ = 4 and *ζ*_*g*_ = 15, which yields the highest correlation with the odor discrimination rates of mice [16]. The anatomy of the olfactory bulb, however, is not consistent with this straightforward result, because the dendrites extending from the granular cells are shorter than those from the mitral cells [32]. However, as the number of granular cells is approximately 100 times that of mitral cells [26], we assumed that the granular cells have a wider influential range than the mitral cells, and searched within the range of 3 ≦ *ζ*_*m*_ ≦ 7, 13 ≦ *ζ*_*g*_ ≦ 17. While this assumption was derived from a previous olfactory model proposed by Linster *et al*. [25], it is also possible that the cortical feedback onto granular cells gives a broader view and suppresses the response of mitral cells in an odor-selective manner. It has long been known that the olfactory cortex sends feedback to the olfactory bulb [33]. The role of this feedback was analyzed by Boyd *et al*. [34], who found that pyramidal cells in the olfactory cortex send odor-selective excitatory feedback to glomerular cells in the olfactory bulb, which gates the response of the mitral cells.

The proposed model and the above discussion are based on the assumption that the suppression of certain activities in the olfactory bulb is the cause of selective adaptation. If this is correct, there are at least two more mechanisms that could be involved in selective adaptation.

1. Intraglomerular inhibition through periglomerular cells
  Interglomerular inhibition can decorrelate similar sensory inputs [35]. Selective adaptation may be a side effect of such a decorrelation.
2. Inhibitory feedback from pyramidal cells in the olfactory cortex to the mitral cells via glomerular cells
  Selective adaptation should involve a learning process and be modulated according to the given task, because successive odor discrimination experiments on mice produced substantially improved discrimination rates for odor [IA EB] against odor [IA EB Ci] [16]. Such task-oriented learning can only be directed by higher brain functions. In addition, the cortical inhibitory feedback to the olfactory bulb helps amplify the odor-evoked inhibition [34], which is an important aspect of selective adaptation. Therefore, cortical feedback is quite possibly involved in selective adaptation and its modulation.
  In addition to the above scenarios, Linster et al. proposed the following mechanism:
3. Synaptic modulation in the olfactory cortex
  Selective adaptation may also be implemented in the olfactory cortex. Combined analysis of computer models and behavior experiments on mice suggests that synaptic adaptation and synaptic potentiation in the olfactory cortex can cause odor-specific habituation [36], which is similar to selective adaptation. (Please note that this study did not discuss odorant mixtures or measure activity patterns.)

Although our simulation results suggest that selective adaptation forms solely in the olfactory bulb, the above possibility should be considered in future to clarify the relationship between selective adaptation and internal representation in the olfactory bulb and olfactory cortex. To achieve this goal, further neural activity data are required from corresponding behavior experiments.

## Conclusion

We have proposed an olfactory model based on previous models [19, 20], as well as some biological facts revealed in previous studies. The proposed model was used to simulate an odor discrimination experiment performed by Takiguchi *et al*. [16], and predicted the perceptual similarity observed in mice. Our results indicate that the proposed model is able to account for selective adaptation in the olfactory system, as observed in the odor discrimination rate of mice. We plan to investigate whether the proposed model is also applicable to humans by clarifying their selective adaptation characteristics, and then improving the proposed model to predict humans’ perceptual characteristics.

## Supporting Information

S1 Appendix Fig A. Odor discrimination experiment using Y-maze. Fig B. Results of odor discrimination experiment. Table A. Glomerular layer. Table B. Respiration function. Table C. Mitral layer and granular layer. Table D. Parameters related to synapse connection matrices. Table E. Dissimilarity evaluation part. Table F. Experimental procedure.
(DOCX)

## References

[1] Atanosova B, Thomas-Danquin T, Chabanet C, Langlois D, Nicklaus S, Etievant P. Perceptual interactions in odour mixtures: odour quality in binary mixtures of woody and fruity wine odorants. Chem Senses. 2005; 30: 209–217. 10.1093/chemse/bji016 15741601

[2] Haddad R, Khan R, Takahashi YK, Mori K, Harel D, Sobel N. A metric for odorant comparison. Nat Methods. 2008; 5(5): 425–429. 10.1038/nmeth.1197 18376403

[3] Khan RM, Luk C-H, Flinker A, Aggarwal A, Lapid H, Haddad R, et al Predicting odor pleasantness from odorant structure: pleasantness as a reflection of the physical world. J Neurosci. 2007; 27(37): 10015–10023. 10.1523/JNEUROSCI.1158-07.2007 17855616 PMC6672642

[4] Soh Z, Tsuji T, Takiguchi N, Ohtake H. An artificial neural network approach for glomerular activity pattern prediction using the graph kernel method and the Gaussian mixture functions. Chem Senses. 2011; 36(5): 413–24. 10.1093/chemse/bjq147 21343242

[5] Soh Z, Saito M, Kurita Y, Takiguchi N, Ohtake H, Tsuji T. A comparison between the human sense of smell and neural activity in the olfactory bulb of rats. Chem Senses. 2014; 39(2): 91–105. 10.1093/chemse/bjt057 24252998

[6] Snitz K, Yablonka A, Weiss T, Frumin I, Khan RM, Sobel N. Predicting odor perceptual similarity from odor structure. PLoS Comput Biol. 2013; 9(9): e1003184 10.1371/journal.pcbi.1003184 24068899 PMC3772038

[7] Haddad R, Medhanie A, Roth Y, Harel D, Sobel N. Predicting odor pleasantness with an electronic nose. PLoS Comput Biol. 2010; 6(4): e1000740 10.1371/journal.pcbi.1000740 20418961 PMC2855315

[8] Mori K, Nagao H, Yoshihara Y. The olfactory bulb: coding and processing of odor molecule information. Science. 1999; 286(22): 711–715. 10531048 10.1126/science.286.5440.711

[9] Sakano H. Neural map formation in the mouse olfactory system. Neuron. 2010; 67(4):530–542. 10.1016/j.neuron.2010.07.003 20797531

[10] Belluscio L, Katz C. Symmetry, stereotypy, and topography of odorant representations in mouse olfactory bulbs. J Neurosci. 2001; 21(6): 2113–2122. 11245695 10.1523/JNEUROSCI.21-06-02113.2001 PMC6762614

[11] Uchida N, Takahashi Y, Tanifuji M, Mori K. Odor maps in the mammalian olfactory bulb: domain organization and odorant structural features. Nat Neurosci. 2000; 3: 1035–1043. 10.1038/79857 11017177

[12] Johnson BA, Woo CC, Leon M. Spatial coding of odorant features in the glomerular layer of the rat olfactory bulb. J Comp Neurol. 1998; 393: 457–471. 9550151 10.1002/(sici)1096-9861(19980420)393:4<457::aid-cne5>3.0.co;2-#

[13] Fletcher ML, Masurkar AV, Xing J, Imamura F, Xiong W, Nagayama S, et al Optical imaging of postsynaptic odor representation in the glomerular layer of the mouse olfactory bulb. J Neurophysiol. 2009; 102: 817–830. 10.1152/jn.00020.2009 19474178 PMC2724327

[14] Youngentob SL, Johnson BA, Leon M, Sheehe PR, Kent PF. Predicting odorant quality perceptions from multidimensional scaling of olfactory bulb glomerular activity patterns. Behav Neurosci. 2006; 120(6): 1337–1345. 10.1037/0735-7044.120.6.1337 17201479 PMC2222860

[15] Grossman KJ, Mallik AK, Ross J, Kay LM, Issa NP. Glomerular activation patterns and the perception of odor mixtures. Eur J Neurosci. 2008; 27(10): 2676–2685. 10.1111/j.1460-9568.2008.06213.x 18445053

[16] Takiguchi N. Performance of mice in discrimination of liquor odors: behavioral evidence for olfactory attention. Chem Senses Advance Access. 2008; 33(3): 283–290. 10.1093/chemse/bjm086 18178544

[17] Goyert HF, Frank ME, Gent JF, Hettinger TP. Characteristic component odors emerge from mixtures after selective adaptation. Brain Res Bull. 2007;72(1):1–9. 10.1016/j.brainresbull.2006.12.010 17303501 PMC1913636

[18] Giraudet P, Berthommier F, Chaput M. Mitral cell temporal response patterns evoked by odor mixtures in the rat olfactory bulb. Neurophysiol. 2002; 88: 829–838. 10.1152/jn.2002.88.2.829 12163534

[19] Li Z, Hopfield JJ. Modeling the olfactory bulb and its neural oscillatory processings. Biol Cybern. 1989; 61: 379–392. 2551392 10.1007/BF00200803

[20] Erdi P, Grobler T, Barna G, Kaski K. Dynamics of the olfactory bulb: bifurcations, learning, and memory. Biol Cybern. 1993; 69: 57–66. 8334190 10.1007/BF00201408

[21] Maresh A, Rodriguez GD, Whitman MC, Greer CA. Principles of glomerular organization in the human olfactory bulb—implications for odor processing. PLoS One. 2008; 3(7): e2640 10.1371/journal.pone.0002640 18612420 PMC2440537

[22] Yamaguchi M, Yamazaki K, Beauchamp GK, Bard J, Thomas L, Boyse EA. Distinctive urinary odors governed by the major histocompatibility locus of the mouse. Proc Natl Acad Sci USA. 1981; 78(9): 5817–5820. 6946517 10.1073/pnas.78.9.5817 PMC348873

[23] Yamazaki K, Beauchamp GK, Singer A, Bard J, Boyse EA. Odortypes: Their origin and composition. Proc Natl Acad Sci USA. 1999; 96: 1522–1525. 9990056 10.1073/pnas.96.4.1522 PMC15502

[24] Hebb DO. The Organization of Behavior: A Neuropsychological Theory. New York: Wiley & Sons; 1949.

[25] Linster C, Menon A V, Singh CY, Wilson DA. Odor-specific habituation arises from interaction of afferent synaptic adaptation and intrinsic synaptic potentiation in olfactory cortex. Learn Mem. 2009; 16(7): 452–459. 10.1101/lm.1403509 19553383 PMC3263734

[26] Naritsuka H, Sakai K, Hashikawa T, Mori K, Yamaguch M. Perisomatic-targeting granule cells in the mouse olfactory bulb. J Comp Neuron. 2009; 515(4): 409–426. 10.1002/cne.22063 19459218

[27] Johnson BA, Woo CC, Leon M. Spatial coding of odorant features in the glomerular layer of the rat olfactory bulb. J Comp Neurol. 1998; 393(4): 457–451. 9550151 10.1002/(sici)1096-9861(19980420)393:4<457::aid-cne5>3.0.co;2-#

[28] Johnson BA et al Functional mapping of the rat olfactory bulb using diverse odorants reveals modular responses to functional groups and hydrocarbon structural features. J Comp Neurol. 2002; 449: 180–194. 10.1002/cne.10284 12115688

[29] Johnson BA, Farahbod H, Leon M. Interactions between odorant functional group and hydrocarbon structure influence activity in glomerular response modules in the rat olfactory bulb. J Comp Neurol. 2005; 483: 205–216. 10.1002/cne.20409 15678471 PMC2222893

[30] Johnson BA, Leon M. Chemotopic odorant coding in a mammalian olfactory system. J Comp Neurol. 2007; 503: 1–34. 10.1002/cne.21396 17480025 PMC2213457

[31] Glomerular response archive. [accessed 7 March 2016]. [Internet]. http://gara.bio.uci.edu/index.jsp

[32] Miyamichi K, Shlomai-Fuchs Y, Shu M, Weissbourd BC, Luo L, Mizrahi A. Dissecting local circuits: parvalbumin interneurons underlie broad feedback control of olfactory bulb output. Neuron. 2013; 80(5): 1232–1245. 10.1016/j.neuron.2013.08.027 24239125 PMC3932159

[33] Haberly LB, Price JL. Association and commissural fiber systems of the olfactory cortex of the rat II. Systems originating in the olfactory peduncle. J Comp Neurol. 1978; 181(4): 781–807. 10.1002/cne.901810407 690285

[34] Boyd AM, Sturgill JF, Poo C, Isaacson JS. Cortical feedback control of olfactory bulb circuits. Neuron. 2012; 76(6): 1161–1174. 10.1016/j.neuron.2012.10.020 23259951 PMC3725136

[35] Cleland TA, Linster C. On-Center/Inhibitory-Surround Decorrelation via Intraglomerular Inhibition in the Olfactory Bulb Glomerular Layer. Front. Integr. Neurosci. 2012; 6(5) 1–10. 22363271 10.3389/fnint.2012.00005 PMC3277047

[36] Linster C, Menon AV, Singh CY, Wilson DA. Odor-specific habituation arises from interaction of afferent synaptic adaptation and intrinsic synaptic potentiation in olfactory cortex. Learn. Mem. 2009; 16(7): 452–459 10.1101/lm.1403509 19553383 PMC3263734

