# Supplementary material for: A Mathematical Model of the Olfactory Bulb for the Selective Adaptation Mechanism in the Rodent Olfactory System
Source: PLoS One. 2016 Dec 19;11(12):e0165230. doi: 10.1371/journal.pone.0165230 (PMC5167254; doi:10.1371/journal.pone.0165230)
Supplement: S1 Appendix — Fig A. Odor discrimination experiment using Y-maze. Fig B. Results of odor discrimination experiment. Table A. Glomerular layer. Table B. Respiration function. Table C. Mitral layer and granular layer. Table D. Parameters related to synapse connection matrices. Table E. Dissimilarity evaluation part. Table F. Experimental procedure. (DOCX) [file pone.0165230.s001.docx]

**S1 Appendix**

**A Mathematical Model of the Olfactory Bulb for the Selective Adaptation Mechanism in the Rodent Olfactory System**

Zu Soh^1*^, Shinya Nishikawa^2)^, Yuichi Kurita^1)^, Noboru Takiguchi^3)^, Toshio Tsuji^1*^

## **Symbols and Parameters**

The parameters and variables used in this paper are listed in Tables A–E. The third column in these tables gives either the parameter value used or “adjustable” if the parameter was adjusted. The symbol “-” denotes a variable.

Table A. Parameters related to glomerular layer

| Symbol | Explanation | Value |
| --- | --- | --- |
| *i* | Number of unit | - |
| *n_q_* | An odorant specified by *q* | - |
| *q* | Odorant number | - |
| *c_i_*(*n_q_*) | Input value corresponding to odorant *n_q_* to *i*-th glomerular unit | - |
| $\boldsymbol{C}\left( n_{q} \right)=[c_{1}\left( n_{q} \right),\ldots,c_{k}\left( n_{q} \right),\ldots,c_{1805}\left( n_{q} \right)]\in\mathbb{R}^{1805\times1}$ | Input vector corresponding to odorant *n_q_*. | - |

Table B. Parameters related to respiration function

| Symbol | Explanation | Value |
| --- | --- | --- |
| *I*_sniff,_*_i_*(t) | Respiration input to *i*-th mitral cell | - |
| *t_i_*_nhale_ | Inhalation duration [19] | 220 [ms] |
| *t*_exhale_ | Exhalation duration [19] | 400 [ms] |
| *τ*_exhale_ | Time constant for exhalation [19] | 33 [ms] |
| *o_γ_* | An odor specified by *γ* | - |
| *γ* | Odor number | - |
| *k* | Odorant number composed in the odor *o_γ_* | - |
| *P_i_(o_γ_)* | Input to *i*-th glomerular unit corresponding to odor *o* | - |
| $\boldsymbol{P(}o_{\gamma}\boldsymbol{)}={[P}_{1}\left( o_{\gamma} \right),\ldots,P_{k}\left( o_{\gamma} \right),\ldots,P_{1805}\left( o_{\gamma} \right)]\in\mathbb{R}^{1805\times1}$ | Input vector corresponding to odor *o_γ_*. | - |
| *θ* | Threshold value for binarization | 0.6 |

Table C. Parameters related to mitral layer and granular layer

| Symbol | Explanation | Value |
| --- | --- | --- |
| *x_L_* | Internal state of mitral unit | - |
| $\boldsymbol{X}=[x_{1},\ldots,x_{k},\ldots,x_{1805}]\in\mathbb{R}^{1805\times1}$ | Internal state vector of mitral units | - |
| *y_L_* | Internal state of granular unit | - |
| $\boldsymbol{Y}=$[$y_{1},\ldots,y_{k},\ldots,y_{1805}]\in\mathbb{R}^{1805\times1}$ | Internal state vector of granular units | - |
| *I_L_* | Input to mitral unit |  |
| $\boldsymbol{I}={[I}_{1},\ldots,I_{k},\ldots,I_{1805}]\in\mathbb{R}^{1805\times1}$ | Input vector to mitral units |  |
| *I*_background_ | Background noise input | 0 |
| *I*_cortex_ | Excitatory input from olfactory cortex | 0 |
| *τ_x_* | Time constant in Eq. (3) [19] | 7 [ms] |
| *τ_y_* | Time constant in Eq. (4) [19] | 7 [ms] |
| *g_x_*(*x_L_*) | Action potential of mitral cell | - |
| $\boldsymbol{G}_{\boldsymbol{X}}={[g}_{x}(x_{1}),\ldots,g_{x}(x_{k}),\ldots,g_{x}(x_{1805})]\in\mathbb{R}^{1805\times1}$ | Action potential vector of mitral cells | - |
| *g_y_*(*y_L_*) | Action potential of granular cell | - |
| $\boldsymbol{G}_{\boldsymbol{Y}}={[g}_{y}\left( y_{1} \right),\ldots,g_{y}\left( y_{k} \right),\ldots,g_{y}\left( y_{1805} \right)]\in\mathbb{R}^{1805\times1}$ | Action potential vector of granular cells | - |
| *S'x* | Constant of asymmetrical sigmoid function (Eq. (5)) [19] | 0.14 |
| *Sx* | Constant of asymmetrical sigmoid function (Eq. (5)) [19] | 1.4 |
| *S'y* | Constant of asymmetrical sigmoid function (Eq. (5)) [19] | 0.29 |
| *Sy* | Constant of asymmetrical sigmoid function (Eq. (5)) [19] | 2.9 |
| *ζ* | Threshold of asymmetrical sigmoid function (Eqs. (5) and (6)) [19] | 1.0 |
| $\boldsymbol{H}={[H}_{(1,1)},\ldots,H_{\left( j,k \right)},\ldots,H_{\left( 1805,1805 \right)}]\in\mathbb{R}^{1805\times1805}$  $\boldsymbol{H}_{\boldsymbol{0}}={{[H}_{0}}_{(1,1)},\ldots,{H_{0}}_{\left( j,k \right)},\ldots,{H_{0}}_{\left( 1805,1805 \right)}]\in\mathbb{R}^{1805\times1805}$ | Synapse connection matrix from granular units to mitral units | - |
| $\boldsymbol{W}={[W}_{(1,1)},\ldots,W_{\left( j,k \right)},\ldots,W_{\left( 1805,1805 \right)}]\in\mathbb{R}^{1805\times1805}$  $\boldsymbol{W}_{\boldsymbol{0}}={{[W}_{0}}_{(1,1)},\ldots,{W_{0}}_{\left( j,k \right)},\ldots,{W_{0}}_{\left( 1805,1805 \right)}]\in\mathbb{R}^{1805\times1805}$ | Synapse connection matrix from mitral units to granular units | - |
| $\boldsymbol{L}={[L}_{(1,1)},\ldots,L_{\left( j,k \right)},\ldots,L_{\left( 1805,1805 \right)}]\in\mathbb{R}^{1805\times1805}$  $\boldsymbol{L}_{\boldsymbol{0}}={{[L}_{0}}_{(1,1)},\ldots,{L_{0}}_{\left( j,k \right)},\ldots,{L_{0}}_{\left( 1805,1805 \right)}]\in\mathbb{R}^{1805\times1805}$ | Synapse connection matrix between mitral units | - |

Table D. Parameters related to synapse connection matrices

| Symbol | Explanation | Value |
| --- | --- | --- |
| *ζ_m_* | Connection range of mitral cells | Adjustable |
| *ζ_g_* | Connection range of granular cells | Adjustable |
| *α_i_* | Position of *i*-th unit on *α-*axis | - |
| *α_A,i_* | Position of the left end of the *α-*axis where *i*-th unit is located | - |
| *β_i_* | Position of *i*-th unit on *β-*axis | - |
| *β_B,i_* | Position of the lower end of the *β*-axis where *i*-th unit is located | - |
| *d_α(i,j)_* | Distance between *i*-th and *j*-th unit on *α*-axis | - |
| *d_β(i,j)_* | Distance between *i*-th and *j*-th unit on *β*-axis | - |

Table E. Parameters related to dissimilarity evaluation part

| Symbol | Explanation | Value |
| --- | --- | --- |
| *S_i,o_* | Activity strength of *i*-th mitral unit responding to an odor *o* | - |
| *E* | Dissimilarity index | - |

## **Odor discrimination experiment on mice**

Takiguchi *et al*. [16] used an 8-week-old mouse (C57BL/6J, male) to perform odor discrimination experiments with a Y-maze behavioral assay. The following describes the experimental results and the selective adaptation function identified by the experiment.

Fig. A shows the Y-maze assay. In the experiments, an individual mouse deprived of water was allowed to run from the start position along either arm, each of which was scented by an air current conducted through an odor box. If the mouse chose the rewarded odor, a drop of water was provided from the water-feeder; otherwise, the mouse was immediately returned to the start position. The mouse performed 24 consecutive trials in one session. The total number of times that the mouse selected the rewarded odor was recorded, from which the discrimination rate was calculated. The experiment essentially performed the following two steps.

Step 1. Operant conditioning training: The rewarded odor and mineral oil (no scent) were conducted into the arms of a Y-maze so that the mouse became conditioned to the rewarded odor.

Step 2. Odor discrimination: The rewarded odor and the discrimination target odor were conducted into the arms of the Y-maze, and the discrimination rate was recorded.

Table F shows the odorant components used in the experiment: the odor set is composed of odorants Isoamyl acetate(IA), Ethyl butyrate (EB), and Citral (Ci).

Table F. Experimental procedure

| Experimental procedure | Rewarded odor | Discrimination target |
| --- | --- | --- |
| Step 1. Learning | [IA, EB, Ci] | Mineral oil |
| Step 2. Discrimination |  | [IA, EB], [IA, Ci], [EB, Ci], [IA], [EB], [Ci] |

Abbreviations: IA: Isoamyl acetate, EB: Ethyl butyrate, Ci: Citral,
An odor is denoted by the components in brackets

Fig. B shows the average discrimination rate of 10 mice with respect to the odor set. The figure demonstrates that most individuals cannot discriminate between the rewarded odor [IA, EB, Ci] and the discrimination target [IA, EB]. This result suggests that the mice focused on the components IA and EB, and eliminated Ci when they were conditioned to the rewarded odor [IA, EB, Ci].

The above results also indicate that selective adaptation is an important factor that should be considered when predicting perceptual similarity.

Fig A. Odor discrimination experiment using Y-maze.


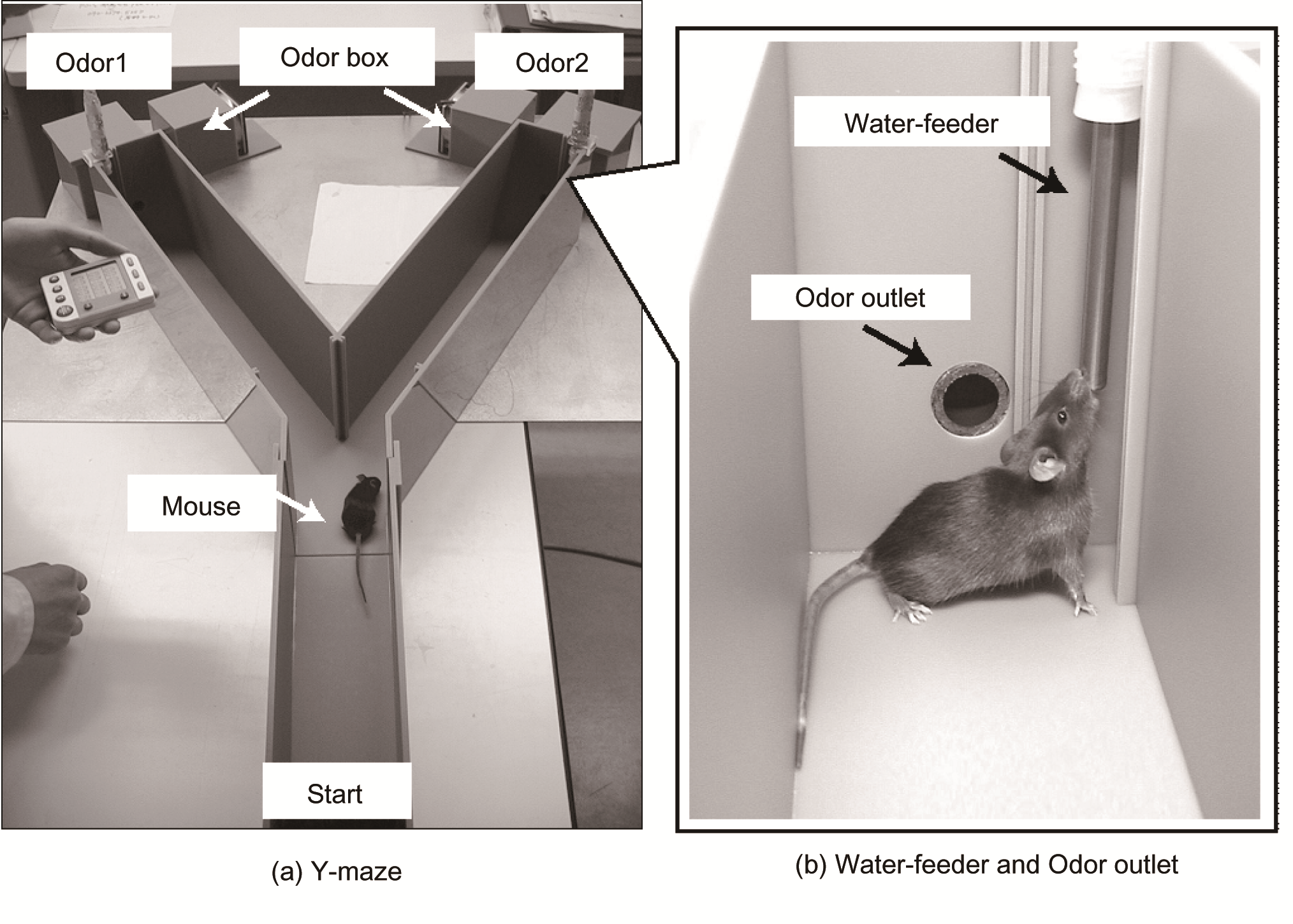

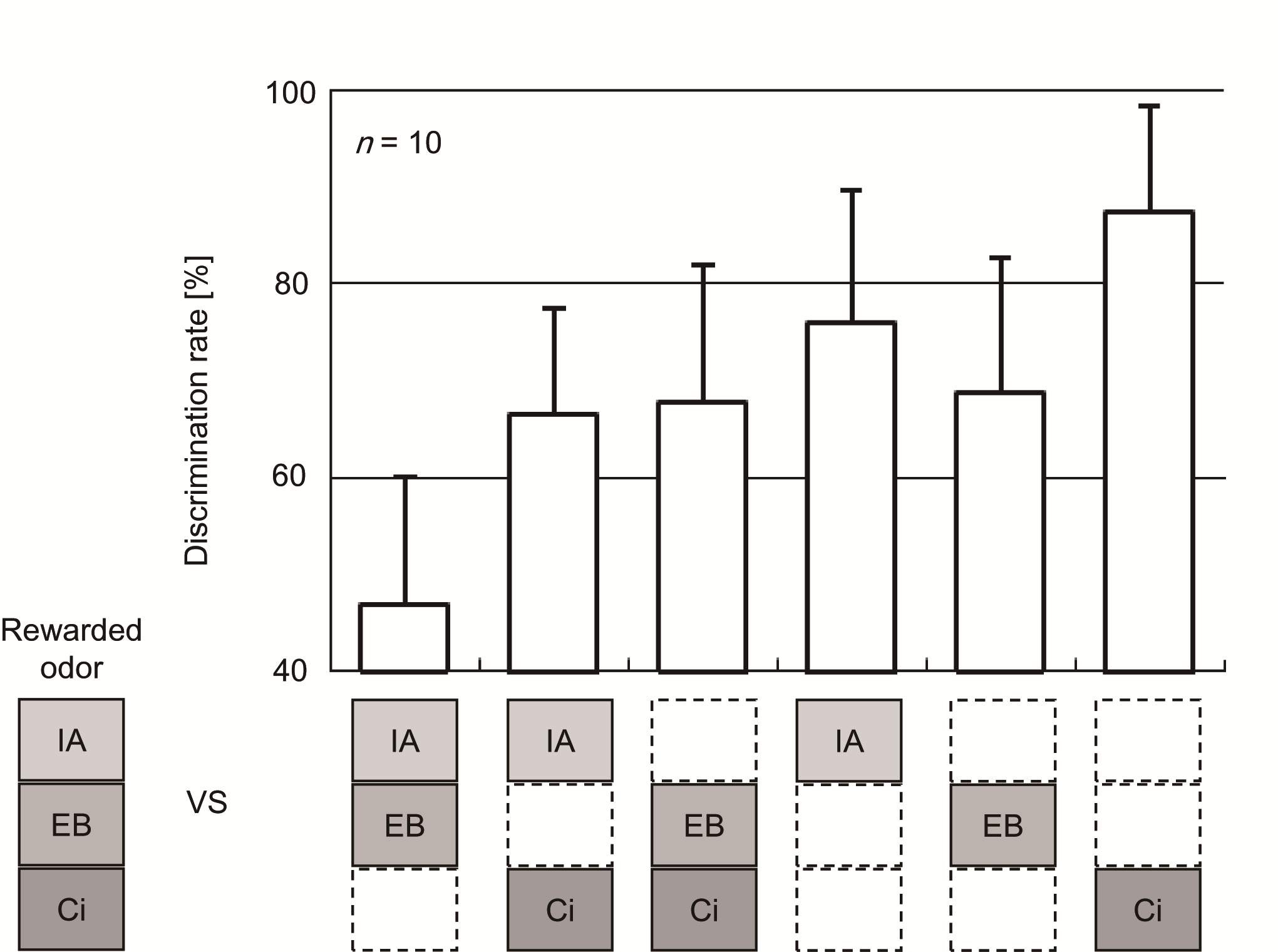


Fig B. Results of odor discrimination experiment
